# Impact of Shared Control Modalities on Performance and Usability of Semi-autonomous Prostheses

**DOI:** 10.3389/fnbot.2021.768619

**Published:** 2021-12-17

**Authors:** Jérémy Mouchoux, Miguel A. Bravo-Cabrera, Strahinja Dosen, Arndt F. Schilling, Marko Markovic

**Affiliations:** ^1^Applied Rehabilitation Technology Lab, Department of Trauma Surgery, Orthopedics and Plastic Surgery, University Medical Center Göttingen, Georg-August University, Göttingen, Germany; ^2^Faculty of Medicine, Department of Health Science and Technology Center for Sensory-Motor Interaction, Aalborg University, Aalborg, Denmark

**Keywords:** prostheses, semi-autonomous, performance, workload, upper-limb

## Abstract

Semi-autonomous (SA) control of upper-limb prostheses can improve the performance and decrease the cognitive burden of a user. In this approach, a prosthesis is equipped with additional sensors (e.g., computer vision) that provide contextual information and enable the system to accomplish some tasks automatically. Autonomous control is fused with a volitional input of a user to compute the commands that are sent to the prosthesis. Although several promising prototypes demonstrating the potential of this approach have been presented, methods to integrate the two control streams (i.e., autonomous and volitional) have not been systematically investigated. In the present study, we implemented three shared control modalities (i.e., *sequential, simultaneous*, and *continuous*) and compared their performance, as well as the cognitive and physical burdens imposed on the user. In the *sequential* approach, the volitional input disabled the autonomous control. In the *simultaneous* approach, the volitional input to a specific degree of freedom (DoF) activated autonomous control of other DoFs, whereas in the *continuous* approach, autonomous control was always active except for the DoFs controlled by the user. The experiment was conducted in ten able-bodied subjects, and these subjects used an SA prosthesis to perform reach-and-grasp tasks while reacting to audio cues (dual tasking). The results demonstrated that, compared to the manual baseline (volitional control only), all three SA modalities accomplished the task in a shorter time and resulted in less volitional control input. The *simultaneous SA* modality performed worse than the *sequential* and *continuous SA* approaches. When systematic errors were introduced in the autonomous controller to generate a mismatch between the goals of the user and controller, the performance of SA modalities substantially decreased, even below the manual baseline. The *sequential SA* scheme was the least impacted one in terms of errors. The present study demonstrates that a specific approach for integrating volitional and autonomous control is indeed an important factor that significantly affects the performance and physical and cognitive load, and therefore these should be considered when designing SA prostheses.

## Introduction

To increase the autonomy of affected users and to meet their requirements (Cordella et al., 2016), upper-limb prostheses have become more dexterous, further enabling the user to perform up to 36 different grasps (i-Limb® Quantum Bionic Hand, Ossur, Reykjavik, Island). However, the standard commercial control based on two-channels and switching was not designed to efficiently accommodate multiple degrees of freedom (DoFs) (Jiang et al., 2012). Myocontrol methods based on machine learning have been investigated for decades (Scheme and Englehart, 2011) to bridge the gap between advanced functionality and poor control. Recently, pattern classification systems have become commercially available (e.g., COAPT engineering and MyoPlus from Otto Bock). They allow users to control several DoFs directly. However, they are sensitive to multiple factors (e.g., muscle fatigue, sweating, and electrode shift), require calibration (retraining), and allow only sequential activation of the DoFs. Regression can be used for simultaneous control, but it can reliably activate only a small number of functions (Hahne et al., 2018, 2020). Finally, machine-learning-based approaches allocate the cognitive burden to the user, who is required to preshape every DoF of the prosthesis to obtain an optimal grasp and avoid compensatory movements.

One approach to improve the control of dexterous prostheses while easing the cognitive burden on the user is to introduce semi-automatic control. This approach is based on enhancing the prosthesis with exteroceptive sensors that allow it to estimate context information. Then, such information can be used to enable the prosthesis to perform certain functions automatically. Semi-autonomous (SA) control was first developed in other fields of assistive robotics (e.g., smart wheelchairs) (Carlson and Demiris, 2012; Novak and Riener, 2015); however, its application in prosthetics is relatively novel. Nevertheless, this approach has recently gained significant momentum both in upper- and lower-limb prostheses (Zhang et al., 2019; Zhong et al., 2021).

Different sensor modalities have been used to automate prostheses during the entire reach-and-grasp sequence. Tactile sensors embedded on the fingers (Tavakoli et al., 2017) help them autonomously adapt to the shape of an object (Zhuang et al., 2019). Inertial measurement units were used to make the prosthesis autonomously react to the orientation of the sound hand in bimanual tasks (Volkmar et al., 2019) or for compensatory movements by rotating the wrist to reduce the need for shoulder elevation (Markovic et al., 2015). A camera, attached to either the prosthesis or to the user's body, can provide information regarding the shape of the object, which can be used to adjust finger aperture, grasp pattern, and wrist rotation (Došen et al., 2010; Markovic et al., 2014, 2015). The RGB data, alone or in combination with depth information, were processed *via* machine (deep) learning to classify objects according to the grasp types that are appropriate for the object (Degol et al., 2016; Fajardo et al., 2018; Hundhausen et al., 2019). In a recent study, computer vision was employed to build a three-dimensional model of the environment while tracking the prosthesis (Mouchoux et al., 2021). This allowed the prediction of points of interaction and automatic preshaping of the hand with an active wrist according to its position relative to the objects in the scene.

Other methods based on sensor fusion were also used to predict reach-and-grasp tasks. They combined gaze tracking with hand tracking (Carrasco and Clady, 2012), electroencephalogram (McMullen et al., 2014), computer vision (Shi et al., 2020), forearm EMG (Krausz et al., 2020), or the movement of a hand-mounted camera (Zhong et al., 2020). Finally, a database of multimodal sensor data was recently published to encourage further development of “intelligent prosthetics” (Cognolato et al., 2020). Importantly, all SA control approaches combine autonomous and volitional control. The former provides “intelligence” to the system, whereas the latter allows the user to gain control when required. However, an excellent method to integrate the two control streams remains an open question, and studies have adopted different *ad hoc* solutions. This question is even more important as there is no guarantee that autonomous controllers will always reliably predict user intention. Several systems implemented “traded autonomy” wherein manual and autonomous controls were activated strictly and sequentially, following an explicit user trigger (Markovic et al., 2014; Fajardo et al., 2018; Volkmar et al., 2019; Mouchoux et al., 2021). More simultaneous approaches were also proposed, where autonomous systems complete the DoFs not controlled by the user (Sherstan et al., 2015) or control all the DoFs of a device if the computed solution agrees with the partial command from the user (Zhuang et al., 2019). However, the effect of these different shared control modalities on the interaction between the user and his/her prosthesis, as well as on the overall performance of the SA system, has not been investigated thus far.

The present study implements three representative shared control schemes (i.e., simultaneous, sequential, and continuous) using the “Wizard-of-Oz” paradigm (Viswanathan et al., 2014; Strazdas et al., 2020). The paradigm is used to compare them in terms of performance and physical and cognitive workload. This approach is often used to study the interaction between a human and a complex or autonomous system. In it, the participant interacts with a computer system that he/she believes to be intelligent, whereas the system in fact “simulates” the intelligence by relying on predefined scenarios and hard-coded interactions. Here, the subjects used an SA prosthesis to conduct reach-and-grasp tasks while reacting to audio cues (i.e., the subject performed a dual task). Specifically, the autonomous controller relied on the “Wizard-of-Oz” approach to automatically and “ideally” adjust prosthesis wrist and hand appropriately for grasping a predefined target object. In some conditions, systematic errors were introduced in the autonomous control module without any knowledge of the subject. This aims to investigate how the shared control approach affects the subject behavior when a mismatch occurs between the intentions of the autonomous controller and the subject.

## Materials and Methods

### System Implementation

**Figure 1:**
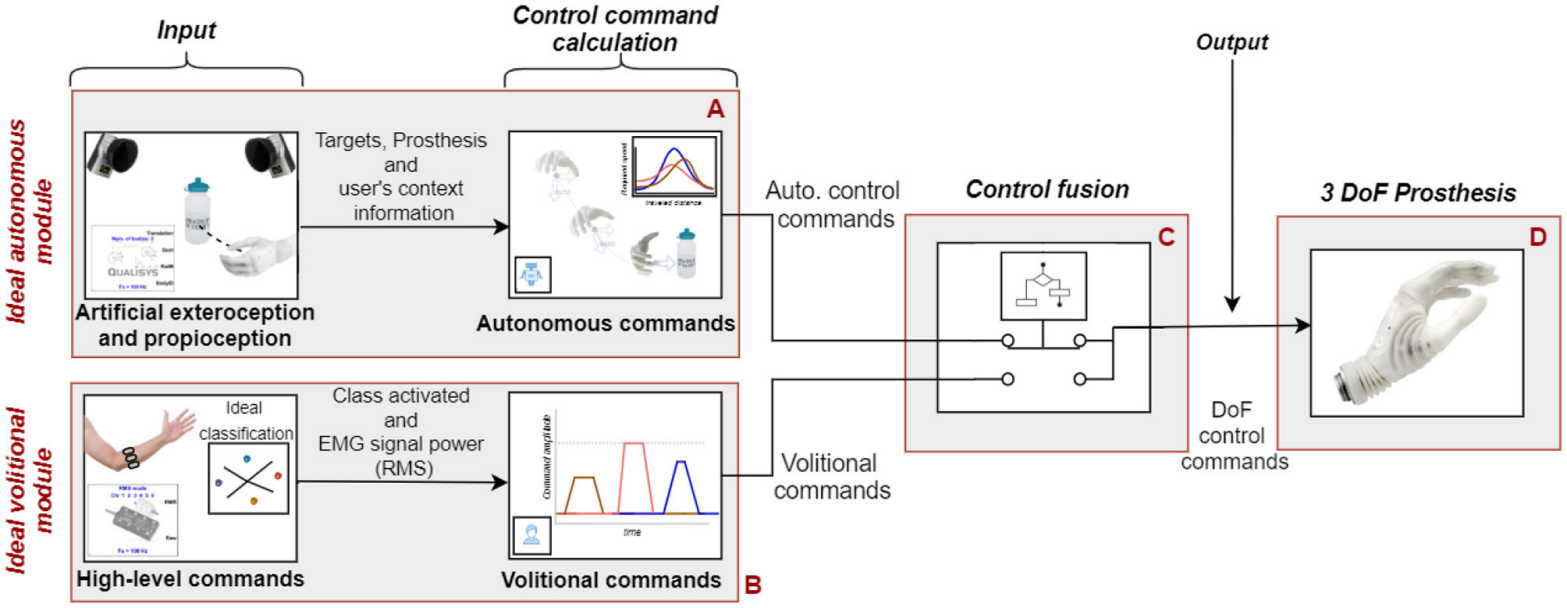
Overall working principle of the implemented SA system. The *ideal autonomous module* **(A)** uses object properties, prosthesis pose tracking (motion capture), and embedded sensor data to calculate control commands to preshape the prosthesis on the fly. The *ideal volitional control module* **(B)** uses an ideal “classification” method (using push-buttons) combined with myocontrol signals to provide sequential and proportional volitional control. The *control fusion module* **(C)** merges the control commands from the two modules into a single command stream to drive the prosthesis DoFs **(D)**. The fusion was performed according to a selected set of rules (shared control modality), as explained in subsection Control Fusion Module.

The SA system (Figure 1) comprises (1) an *ideal volitional control module* that implements sequential and proportional control of multiple DOFs, (2) an *ideal autonomous control module* built using a state-of-the-art motion capture system, and (3) *a control fusion module* that integrates the decisions from the two control streams (i.e., the volitional and the autonomous modules) to produce the final command that was transmitted to a prosthesis. The “ideal” modules mimicked the state-of-the-art autonomous (computer vision; Mouchoux et al., 2021) and volitional [pattern classification using EMG (Iqbal et al., 2018)] control. However, they were implemented using a reliable and well-controlled setup. The Wizard-of-Oz paradigm was adopted because the focus of the study was on the subject behavior and interaction with the system. Hence, the aim was to avoid confounding factors related to the performance of specific implementations.

Both the autonomous and volitional control modules were capable of driving all three DoFs (i.e., rotation, flexion/extension, and palmar open/close) of the left-hand prosthesis (Michelangelo, Ottobock, Austria). The control fusion module processed volitional and autonomous commands according to one of the predefined shared control schemes, as explained in Subsection Control Fusion Module.

#### Ideal Volitional Control Module

**Figure 2:**
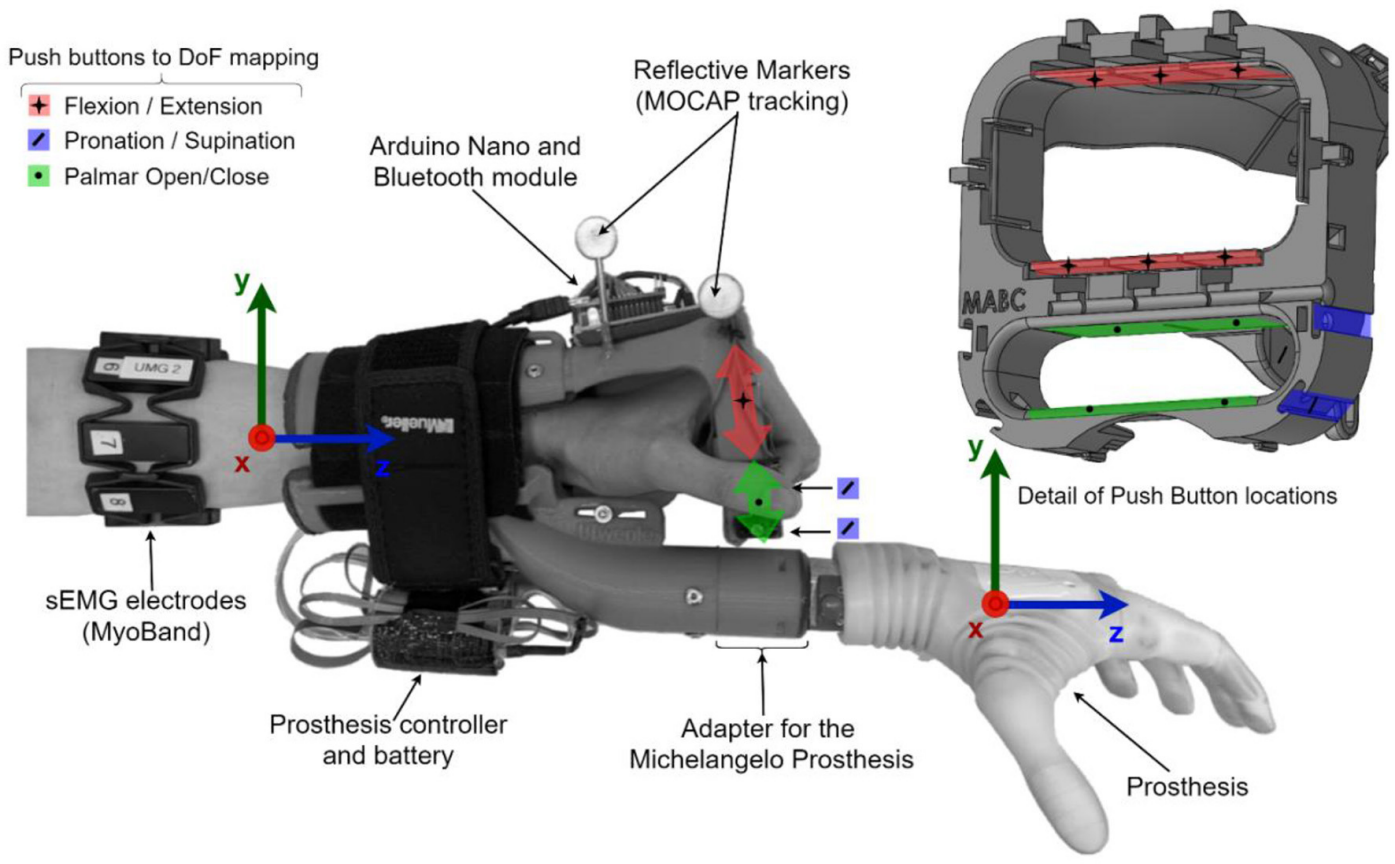
Sensorized prosthesis adapter. The left-hand Michelangelo prosthesis was attached to a three-dimensional printed adapter fixed on the left forearm of the able-body participant. Three infrared markers enabled the tracking of the adaptor in space. Push buttons were embedded in the adaptor around the hand, under the fingers, and under the thumb to detect movement patterns associated with a specific prosthesis DoF. Eight surface EMG electrodes provided proportional control.

This module comprises (1) a custom-made mechanical adapter to map the subject's hand and wrist movements into the prosthesis functions (DoF selection) and (2) a myoelectric (EMG) armband to provide a proportional control signal (see Figure 2).

The state-of-the-art approach in myocontrol is to use the EMG pattern classification to estimate the subject motion intention and map it into the prosthesis functions. This method allows intuitive control, but is also prone to misclassifications. To implement a reliable command interface, we did not rely on interpreting the myoelectric activity; instead, a custom-made adapter was produced to fully encompass the subject's hand and detect the movement intention *mechanically*. The adapter identified the movements through push-buttons triggered by performing selected gestures (wrist flexion/extension, thumb triggering, and fingers opening/closing), as illustrated in Figure 2. Hence, it allowed the user to reliably select the desired *class* by physically performing a given hand motion. The push-buttons were connected to an Arduino Nano board that streamed their states to the host PC *via* a Bluetooth connection at 100 Hz.

In addition, the myoelectric activity was recorded using eight surface EMG electrodes placed around the forearm (Myoband, Thalmic Labs, Inc., Waterloo, Canada) to implement a proportional speed control of the selected DoF. The Myoband streamed the EMG data at 200 Hz to the host PC *via* a Bluetooth connection. To smoothen the signal, the root mean square (RMS) of EMG was computed over 150 ms windows with an overlap of 10 ms between consecutive windows. Then, the RMS from the eight EMG channels was averaged to estimate the overall magnitude of muscle activation along the forearm. The average RMS was normalized to 80% of the maximum voluntary contractions (MVC) and mapped to the normalized movement speed of the selected DoF.

#### Ideal Autonomous Module

**Figure 3:**
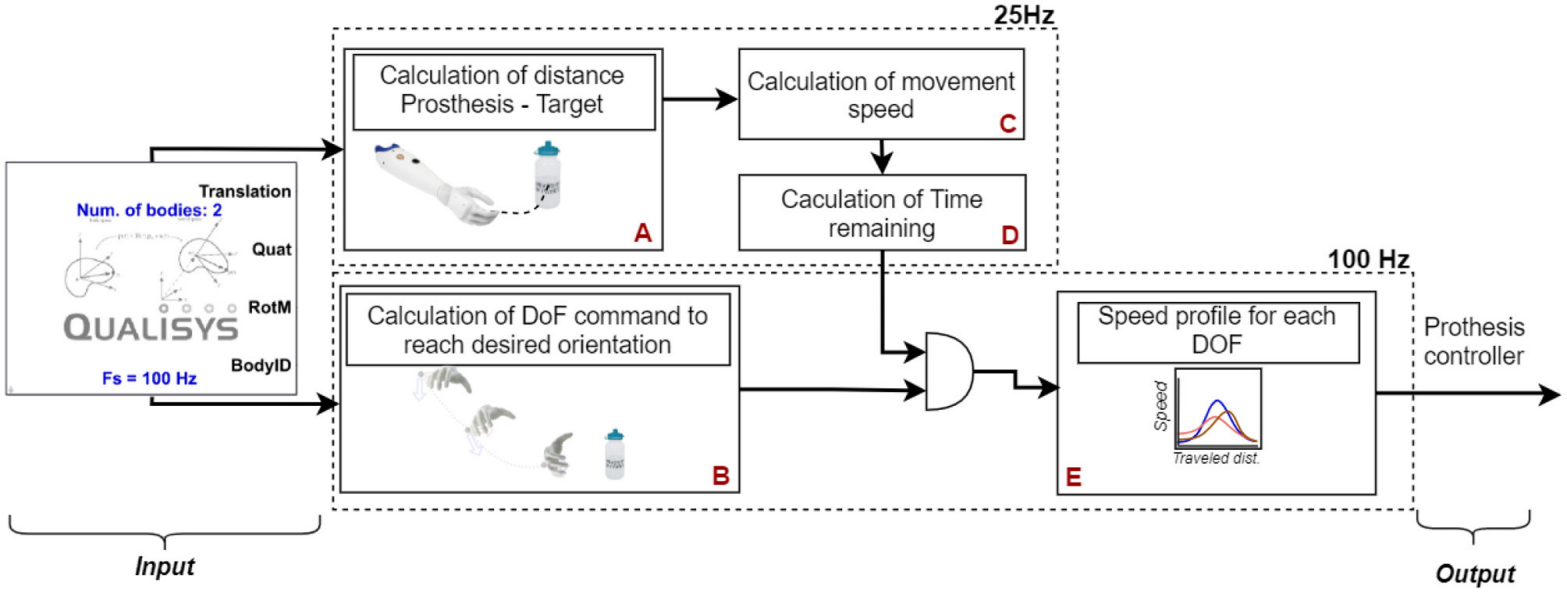
Components of the ideal autonomous module. The tracking information from the motion capture system was used to calculate the current distance from the prosthesis to the target object **(A)**, as well as the difference between the current prosthesis orientation and the desired grasping orientation **(B)**. The module calculated the derivative of the prosthesis position to determine its movement speed **(C)**. The time remaining to reach the target object was computed from the movement speed and current distance to the target object **(D)**. Then, the remaining time (output of **D**) was combined with the difference between current and desired configuration (output of **B**) to compute the speed required for each DoF to reach its target pose at the end of the reaching movement, to accompany the movement of the forearm **(E)**.

The ideal autonomous control system automatically preshaped the prosthesis' wrist (rotation and flexion/extension) during the object reaching phase and simultaneously maintained the hand of the prosthesis opened at 90% of the full aperture. To this end, the module used prosthesis position, orientation, and internal states (i.e., artificial proprioception), along with the position and orientation of the target objects (i.e., artificial exteroception) to calculate the commands for each prosthesis DoF (Figure 3). Typically, the pose and orientation of the target object can be estimated using an RGB sensor (Markovic et al., 2014) or an RGB-D sensor (Mouchoux et al., 2021). However, the present study aimed to obtain reliable control; hence, these parameters were predefined. Similarly, the *target object's desired grasping configuration* (flexion/extension and rotation) was calibrated according to the user preference at the beginning of each session, as explained in subsection Experimental Protocol.

The velocity of each prosthesis DoF was set proportional to the speed at which the subject moved the lower arm (to which the prosthesis was attached) toward the target object, thereby mimicking the smoothness of natural movements during pre-shaping (Jeannerod, 1984). To implement this in a robust and reliable (i.e., *ideal*) manner, the artificial exteroception block employed a state-of-the-art motion capture system (Qualisys Ltd., Gothenburg, Sweden), which tracked the prosthesis and pose of the target objects and streamed it at 100 Hz. Similarly, the artificial proprioception was achieved using (1) the motion tracking to infer the prosthesis position and orientation in space, (2) prosthesis position encoders to retrieve the state (angle) of each prosthesis DoF, and (3) the pressure sensor of the prosthesis thumb to detect contact with the target object. The tracking information (i.e., speed and direction) was extrapolated using quadratic splines based on the data from the previous 2 s to compensate for the occasional marker occlusions.

**Figure 4:**
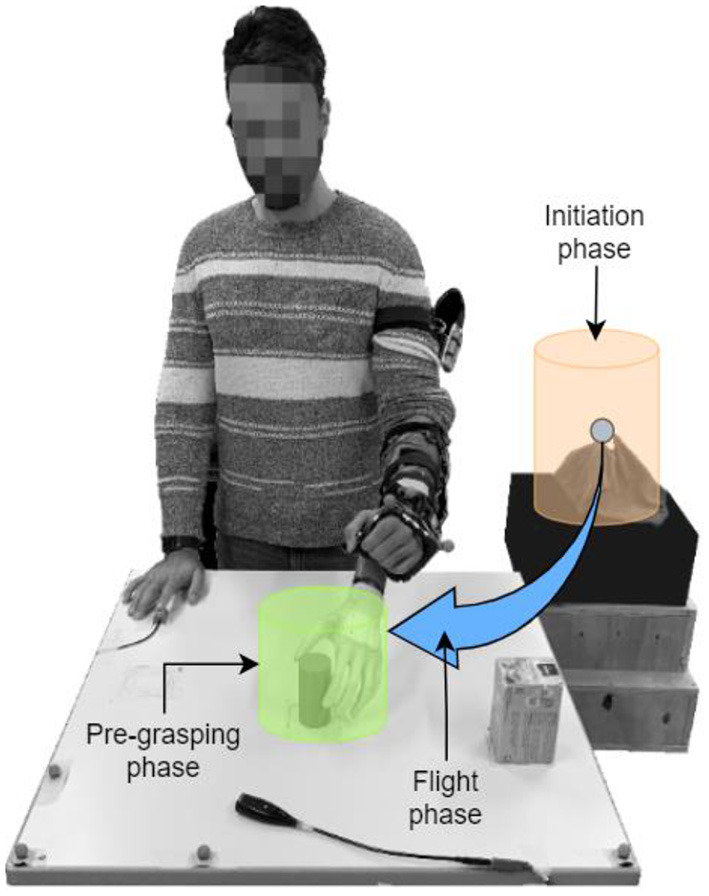
Phases of pre-shaping. The three phases of pre-shaping are defined as the ratio of the distance traveled by the prosthesis to the total distance. The distance of [0, 15]%, [15, 75]%, and [75, 100]% of the total distance corresponds to initiation, flight and pre-grasping phase, respectively.

To achieve gradual progression from the initial to the final stage of the prosthesis's configuration, the module divided the travel distance of the prosthesis from the starting point (see Section Experimental Setup and Protocol) to the target object position into three phases (Figure 4): (1) initiation, (2) flight, and (3) pre-grasping. The movement's progress at any given time was defined as the ratio of the distance traveled by the prosthesis to the total distance. When the travel distance was below 15%, the movement was in its initiation phase, and hence the autonomous module was disabled. This stabilized the behavior of the system by preventing the autonomous controller from abruptly reacting to a small arbitrary movement of the user, unrelated to the reaching motion. By initializing the autonomous control only when the 15% of the traveled distance has been reached, the system gave the impression to the user that it has actually reacted to his/her grasping intention. Since during natural prehension movement, the wrist and fingers reach their final position at ~75% of the movement distance, the flight phase was set to last from 15 to 75% (Jeannerod, 1984). During this phase, the system activated the prosthesis DoFs to ensure that they reached the final state at the end of the flight phase (75%). To do so, the DoF speed was set based on the estimated remaining time to complete the flight phase (Figure 3E):


(1) $$\begin{array}{r} Speed_{DoF}=\;Forearm_{speed}\;\times \frac{\left|Pose_{Final}-Pose_{Current}\right|}{Dist_{total}-Dist_{traveled}}, \end{array}$$

where *Pose*_*Final*_ is the normalized final DoF angle; *Pose*_*current*_ is the normalized current DoF angle; *Dist*_*total*_ and *Dist*_*traveled*_ are the total and traveled distances, respectively; and *Forearm*_*speed*_ is the speed at which the prosthesis moved toward the target computed as the derivative of its position.

Finally, in the *pre-grasping* phase, the autonomous system compensated for the subject's variations in the limb position to maintain the *desired grasping configuration*, which minimized the difference between the *desired grasping configuration* and the actual prosthesis state at any given time, further ensuring a steady pose of the hand with respect to the target object regardless of the forearm movements. The orientation (represented in quaternion) of the prosthesis (*Q*_*Prosth*_) (Equation 2) was computed using the orientation of the forearm (*Q*_*forearm*_) and the current state of the prosthesis wrist DoF according to the coordinate system of the prosthesis socket (*Q*_*Prost*_*h*__*wrist*__).


(2) $$\begin{array}{r} Q_{Prosth}=Q_{forearm}*\left(Q_{forearm}*Q_{Prosth_{wrist}}*Q_{forearm}^{-1}\right) \end{array}$$

The current orientation of the prosthesis (*Q*_*Prosth*_) was then compared to the *desired grasping configuration (Q*_*DesiredGraspConf*_) to determine the difference in orientation that had to be compensated *(Q*_*Compensation*_) (Equation 3).


(3) $$\begin{array}{r} Q_{Compensation}=Q_{DesiredGraspConf}*\left(Q_{Prosth}*Q_{forearm}*Q_{Prosth}^{-1}\right) \end{array}$$

Then, the compensated orientation *(Q*_*Compensation*_) was transformed into a three-angle representation, and the autonomous control module activated each DoF accordingly. The actuation speed in this phase was set to the maximal speed (78°/s in rotation and 90°/s in flexion). The prosthesis DoFs were driven at their maximum speed to ensure that the required orientation was attained at the earliest to ensure that the prosthesis was prepared for the imminent grasping action. The movements in this phase were typically triggered if the automatic controller did not have enough time to fully complete the preshape during the preceding flight phase and/or if the subject performed unexpected movements (e.g., “wobbling” around the object).

#### Control Fusion Module

**Figure 5:**
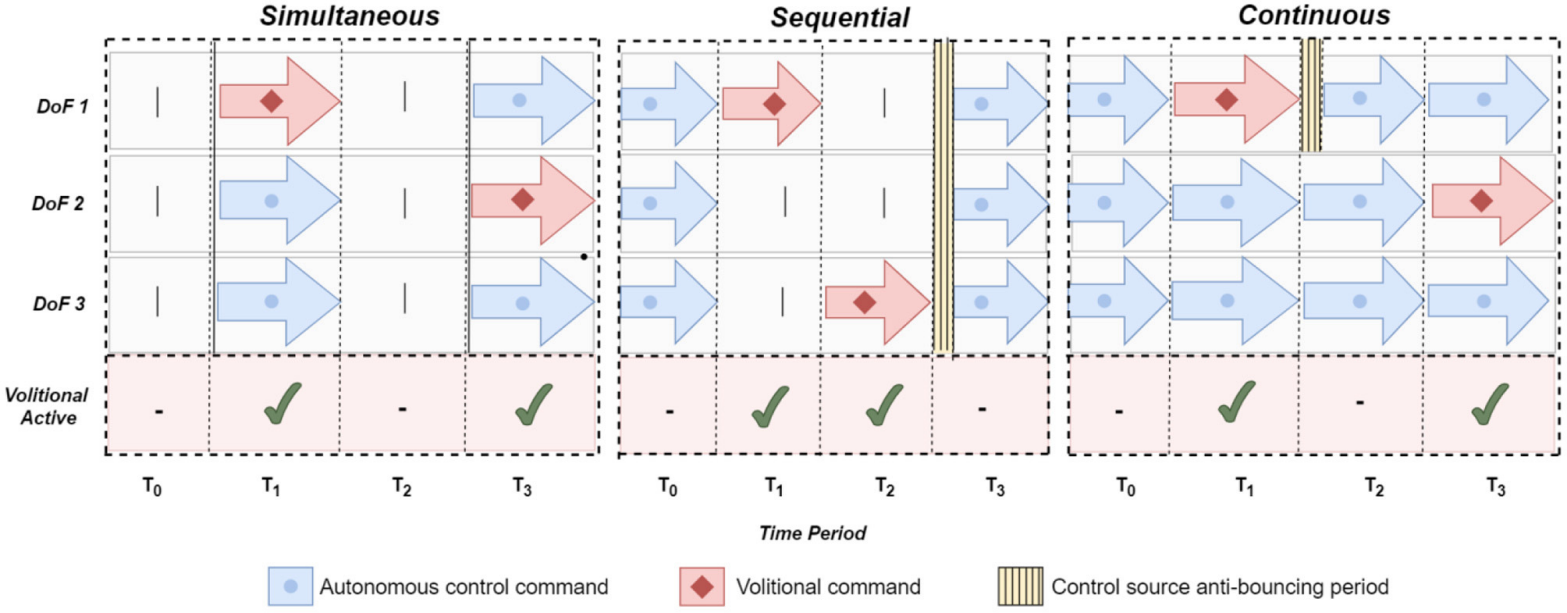
Shared control modalities. Three different approaches to fuse volitional and automatic control tested in this study: *sequential, simultaneous*, and *continuous* (see text for details). The horizontal axis (columns) represents the consecutive time periods. The switch from manual control to automatic control is always proceeded by an off period to avoid oscillating between the control sources.

Three different shared control schemes were implemented (Figure 5): *simultaneous SA, sequential SA*, and *continuous SA*.

In the *simultaneous SA* scheme, the autonomous system complemented the subject's actions only while the user actively controlled the prosthesis using a volitional control interface. Whenever a DoF was controlled manually, the autonomous system would control the remaining DoFs. For example, while the user controls the prosthesis aperture, the autonomous module adjusts its wrists.

In the *sequential SA* scheme, the autonomous system controlled the prosthesis only while there was no volitional input. Unlike the previous approach, the autonomous module was deactivated when the subject controlled the prosthesis aperture. After the volitional input was stopped, the autonomous module resumed the control of all the prosthesis DoFs. To avoid the oscillation between two control modalities, the autonomous system re-activated 1.5 s after the last volitional input.

In the *continuous SA* scheme, the autonomous system *continuously* controlled all DoFs, but relinquished the DoF actuated volitionally by the subject. Therefore, if the subject controlled the flexion/extension, the autonomous module drove the rotation. When the subject stopped generating volitional commands, the prosthesis wrist was fully controlled by the autonomous module. As in the *sequential* scheme, the control was switched to autonomous after a delay of 1.5 s.

### Experimental Setup and Protocol

The experiments were performed in 10 able-bodied subjects (five females and five males), within the age group of 24–55 years, all right-handed. All subjects signed a written consent form approved by the ethical committee of the University Medical Center Göttingen (22/04/16).

The experiment involves reach-and-grasp tasks wherein the subject received specific instructions regarding the techniques by which an object can be grasped using the left-hand prosthesis while reacting to acoustic stimuli (see Subsection Experimental Task).

#### Experimental Task

**Figure 6:**
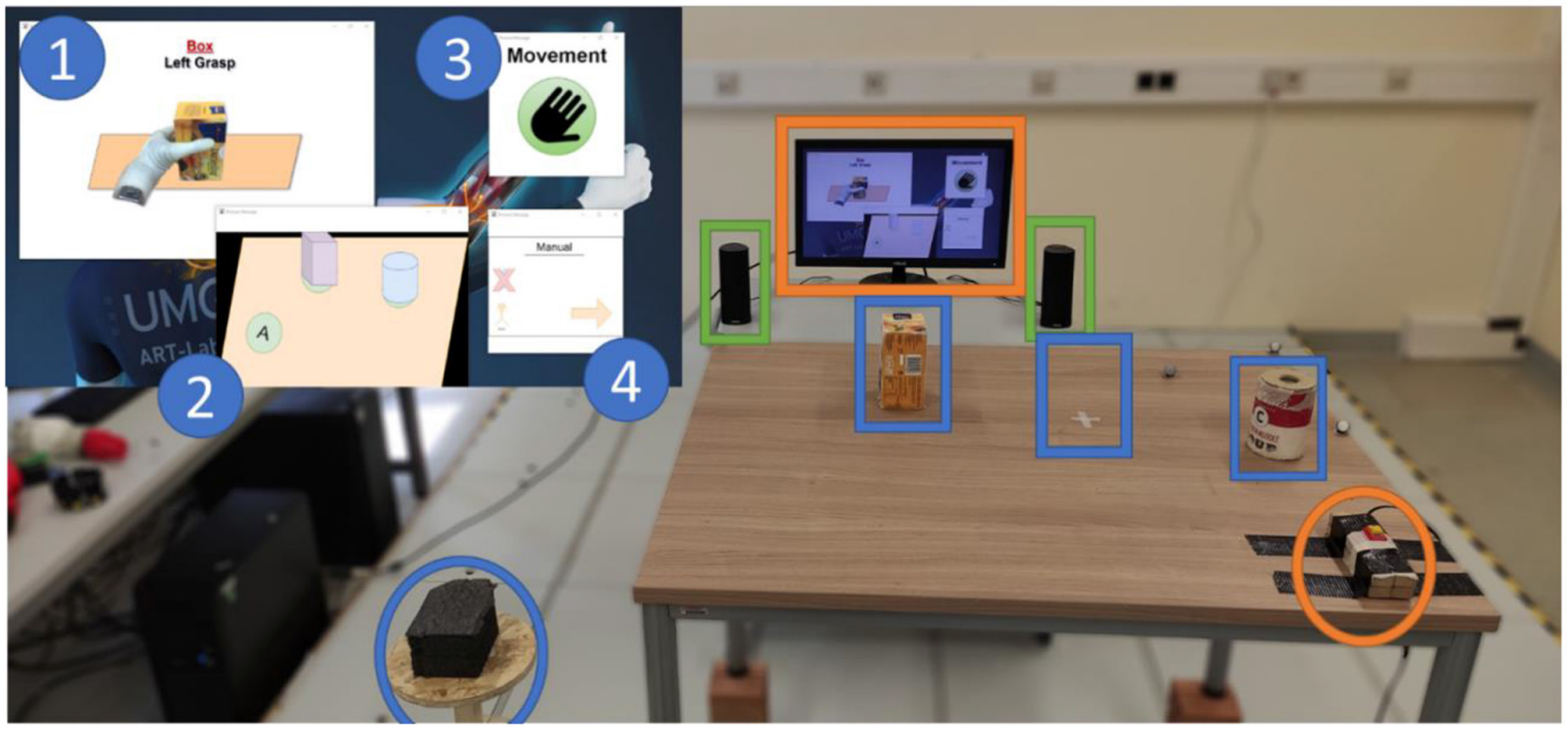
Experimental setup. One rectangular tee box and one cylindrical container were placed on the table in two of the three marked positions (blue squared). The participant started the trial with the prosthesis placed on the rest area (blue circle) and grasped the object as shown on the screen (orange square). In parallel, the participant had to react to each auditory cue delivered by the speakers (green square) by pressing the button on his/her right (orange circle). The screen displayed (1) the instruction on which object should be grasped (box or cylinder) and how the grasp should be performed (from the right, left, top, and front), (2) how the table surface should look like at the end of the manipulation with the object, (3) the visual countdown for the start of the trial indicating when the subject should start reaching toward the object, and (4) the current SA control modality.

The experimental setup from the subject's perspective is depicted in Figure 6. The subject stood in front of a table whose height was adjusted to ensure that her elbows would be at an angle of 90° when the hands were resting on it. Two objects, a box and a cylinder, were placed on the table in two of the three marked positions, as illustrated in Figure 6. A monitor displayed the instructions in front of the subject, i.e., the target object and grasping side. A platform placed on the left of the participant was utilized as a support for the forearm at the initial (starting) position and served as the resting position to reduce muscle fatigue. After a 3-s countdown that is displayed on the screen, a visual signal was provided to the participant to set the beginning of the trial. The participant was required to reach for and grasp the target object from a specific side (right, left, top, and front) as fast as possible and to reallocate it to the (only) available marked position on the table, thus rearranging the setup in each trial. After the target object was allocated, the participant was instructed to move back her arm with the prosthesis to the initial (starting) position and place it back on the platform. Note that the automation was active only during the reaching phase, and hence the experimental task included only reaching, grasping and simple relocation of the object (i.e., object manipulation was not relevant).

During the execution of the reach-to-grasp task, a pseudo-randomly occurring acoustic stimulus (a continuous monotonous beep of 400 Hz) was played through the computer speakers. Each stimulus was played continuously until the participant reacted to it by pressing a button that was fixed on the right side of the table with her right hand, that is, the hand that was not used for prosthesis control. After the participant had reacted to the auditory stimulus, a new cue appeared after a time interval that was randomly selected to last between 0.8 and 1.6 s. The audio cues continued to (re)play until the user grasped the target object.

#### Experimental Protocol

The experiment started by calibrating the SA system to match the individual preferences of each subject, including (1) the responsiveness of each DoF of the prosthesis to volitional input and (2) the prosthesis *desired grasping configuration* during the reach-to-grasp task.

To calibrate volitional control, the participant was asked to trigger each movement using maximal contraction (MVC), followed by a light contraction (activation threshold). The myoelectric signal range between the measured activation threshold and 80% MVC for the given movement was then mapped to the full range of prosthesis velocity for that DoF. Finally, the subjects were asked to perform 20 grasps using the ideal volitional interface to familiarize themselves with it. During these interactions, minor adjustments were performed in control responsiveness as per their requirements.

The subject was asked to control the prosthesis using a volitional interface to grasp both target objects placed on one of the marked positions for every grasping side (right, left, top, and front) to adjust the desired grasping configuration. The states of the prosthesis DoFs (flexion/extension and rotation) and the orientation of the subject's forearm at the moment of contact were recorded as the *desired grasping configuration* for the given object and grasping side. This was repeated for all combinations of target objects and grasping sides, and a lookup table of the *desired grasping configurations* was created. The autonomous module used the lookup table to set the final flexion/extension and rotation (see Subsection Ideal Autonomous Module).

After calibration, the subject started the experimental task (described in Subsection Experimental Task). Before commencing each trial, the two DoFs of the prosthetic wrist were rotated 80° from the final position in a random direction (flexion, extension, rotation, or supination). Such random alterations in the initial prosthesis' orientation were used as a pragmatic solution to enforce a multitude of different pathways that the prosthesis had to transverse in order to reach its final orientation, without having to resort to the (tedious) reorientation of the target object itself. Indeed, the combination of four possible object grasp-directions (left, right, top, or front) and four possible perturbations in the prosthesis' initial orientation (wrist flexion, extension, rotation, or supination) yielded 16 unique combinations in the orientation of the prosthesis at the beginning and end of the trial (i.e., between its initial and final orientation). The subjects performed reach-to-grasp tasks using three SA control schemes (*simultaneous SA, sequential SA*, and *continuous SA*). Each scheme was employed under three conditions, in which the autonomous controller navigated the hand to the *desired configuration* with no error (SA baseline) and with moderate and large systematic errors, respectively. The systematic error was implemented as a constant offset added to the decisions of the autonomous controller, 30° for moderate and 60° for large errors. Additionally, the subjects performed the experimental task using the ideal volitional control (manual baseline), resulting in 10 conditions.

Ten conditions were performed in two blocks of twelve trials each (24 trials per condition). The subjects performed the blocks in a random order. The shared control modality was announced to the subject each time it changed. A break of 10–15 min was made in every six blocks; hence, there were three breaks in total.

### Outcome Measures and Data Analysis

The following outcome measures were employed: (1) the duration of the trial as a measure of performance, (2) the usage of volitional control quantifying the “amount” of a subject's intervention during the task (physical workload), and (3) the user's reaction time to auditory cues as a measure of their cognitive load. Trial duration was defined as the duration between the start signal and the moment of contact with the target object (grasp onset). Volitional control usage was calculated as the accumulated time of volitional control throughout the trial. Finally, the reaction time was defined as the period from an acoustic stimulus until the subject pressed the button.

The first two trials of each block were regarded as an adaptation to a new condition, and these were discarded from the analysis. Therefore, out of 240 performed trials, 200 were used for the data analysis. For each condition, the average values of the outcome variables were computed for each participant. Because none of the outcome variables passed the Kolmogorov-Smirnov test, the difference between the conditions was assessed at first using the Friedman test, and thereafter, in the case of a positive result, using the Wilcoxon signed-rank test for realizing the pairwise comparison. To account for a large number of statistical tests, the *p*-values were corrected using the Bonferroni-Holm correction. The results in the text are reported as median (interquartile range).

## Results

**Figure 7:**
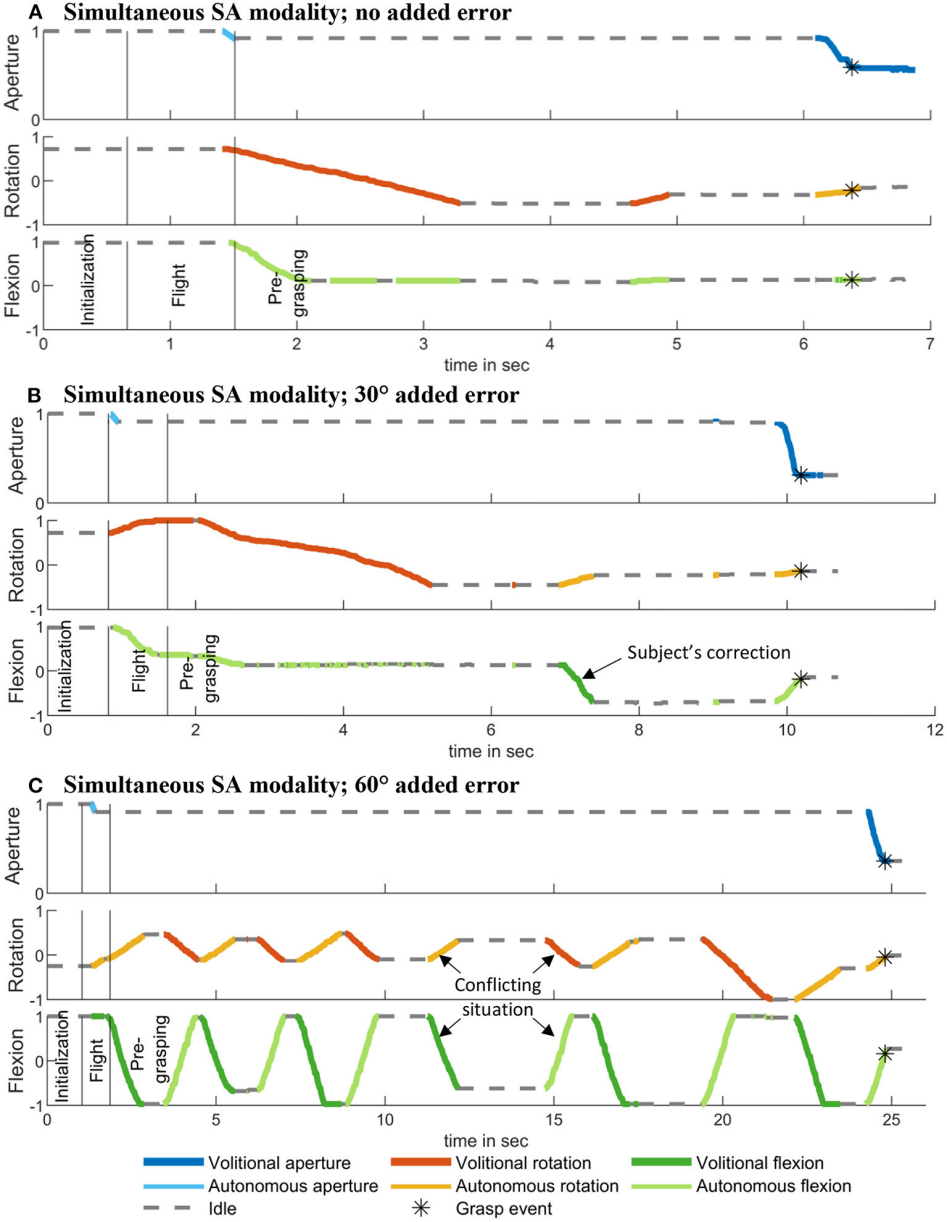
Examples of prosthesis pose during the simultaneous SA modality under 0°, 30°, and 60° added error. Movements due to the volitional control are dark, while those from autonomous controller are light. The bars marking the different phases of the reaching movement correspond to 15 and 75% of the movement. The detection of the grasp is marked with a star. **(A)** Simultaneous SA modality; no added error. **(B)** Simultaneous SA modality; 30° added error. **(C)** Simultaneous SA modality; 60° added error.

During the experiment, volitional and autonomous controllers shared the control of the prosthesis. The pose of the different DoFs of the prosthesis during three trials (i.e., simultaneous no error, simultaneous 30° error, and simultaneous 60° error) is illustrated in Figure 7. Per design, the simultaneous SA modality only allowed the autonomous controller to control the prosthesis when the user actuated one of the prosthesis DoFs. When no error was added to the output of the autonomous controller, the actuation of one DoF was sufficient to preshape the entire prosthesis owing to the autonomous controller complementing the preshape simultaneously (Figure 7A). When 30° errors were added, adjustments were occasionally necessary. The solution provided by the autonomous controller was not sufficient to directly grasp the object, and a short volitional correction was required to rectify it (Figure 7B). At the maximum level of added error, a conflicting situation appeared where the autonomous controller moved back a DoF previously corrected by the user (i.e., the race condition). This produced repetitive corrections of two DoFs until a graspable prosthesis's pose was found (Figure 7C). For the three error conditions, most of the actuation was performed in the vicinity of the object.

**Figure 8:**
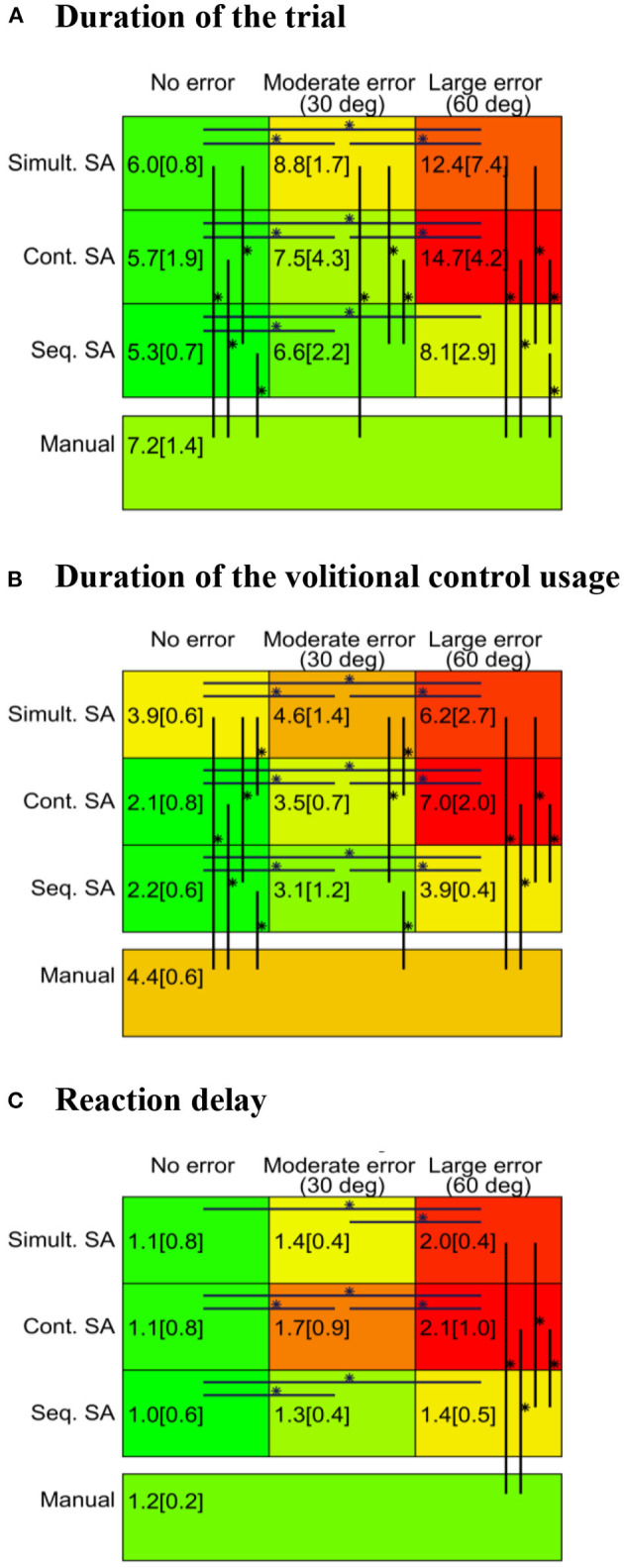
Outcome measures. Duration of the trial **(A)**, duration of the volitional control usage **(B)**, and reaction delay **(C)** for three different SA control modalities (in three different conditions) and the manual control. The depicted values are the medians (interquartile ranges) of the calculated averages for all participants in the respective condition. The green-to-red color scale is normalized to the minimal and maximal values of each outcome measure, respectively. “*” indicates significant difference at the *p*-value lower than 0.05 after the correction.

Subjects grasped the cylinder and the box object equal number of times (100; ten times per condition) and the grasp distribution of the four grasping directions was uniform - 50 grasps were performed from the left, right, top, and front sides of the two objects. On average, per condition, there were 9.9 unique combinations in the orientation of the prosthesis at the beginning and end of the trial (see section Experimental Protocol). A summary of the results for all outcome measures across different conditions is depicted in Figure 8, where the rows are the control modalities and the columns are the error conditions. Statistically significant differences are indicated by horizontal (across error conditions) and vertical lines (across modalities). Regarding the time required to complete the task (Figure 8A), the use of the SA control in the no-error condition always resulted in lower task completion times than manual control independent of the SA control modality. In the no-error condition, differences appeared between the SA modalities, as indicated by better performance obtained when using *sequential SA* compared to *simultaneous SA* modality. The addition of systematic errors impacted all three modalities by increasing the task completion time, but the increase was not equal across the modalities. Moreover, the difference in performance between the modalities became more pronounced as the error increased. The increase in task completion time [median (interquartile range)] was significantly higher (*p* < 0.05) for *simultaneous SA* [+6.3 (6.3) s] and *continuous SA* [+9.1 (3.9) s] compared to the *sequential SA* modality [+3.1 (1.5) s]. Overall, the *sequential* control was faster than *simultaneous SA* and *continuous SA* modalities in both moderate and large error conditions. This increase in the time required to complete the task when using SA control corrupted by the systematic error also impacted the comparison with the manual control baseline. In the moderate error condition, the *simultaneous* modality demonstrated worse time performance than the manual baseline, while in the large error condition, all modalities were slower than the manual baseline.

Volitional control usage exhibited similar trends compared to those observed for the trial duration (Figure 8B). Compared to the manual condition, the use of SA control with no errors consistently reduced the need for volitional control. However, the increase in the error level increased the use of volitional control for all the three SA modalities. Consequently, the volitional control usage in *simultaneous SA* and *continuous SA* modalities became similar in the moderate error condition and eventually longer in the large error condition compared to the manual baseline. However, the sequential modality resulted in a reduced volitional control usage with respect to the baseline in both no error and moderate error conditions and a similar duration in large error conditions. When comparing the SA modalities to each other, the *simultaneous SA* modality required more volitional control in no-error and moderate error conditions than the two other modalities, which were similar to each other. In large error conditions, *sequential SA* modality required less volitional control compared to the other two similar modalities. From moderate to large error conditions, the use of volitional control increased significantly more (*p* < 0.05) for the *continuous SA* [+3.4 (2.0) s] than for the *sequential* SA modality [+0.7 (0.9) s].

The subject's reaction time (Figure 8C) was similar in the no-error and moderate-error conditions across all control approaches (SA and manual). Nevertheless, all modalities demonstrated an increase in the reaction time with an increasing level of error. The *continuous SA* modality seemed to be the most sensitive as the reaction time was different for each combination of error conditions. For *the sequential SA* scheme, the time increased in large and medium error conditions with respect to no-error, whereas for the *simultaneous SA* approach, only the large error condition increased with respect to time. Across the modalities, the only difference was registered for the large error condition, where *sequential SA* control resulted in the smallest time, similar to that achieved in the manual baseline. The reaction time in the other two modalities were higher than those in the manual baseline.

## Discussion

We investigated three different schemes for the integration of volitional and autonomous control in a SA prosthesis. To avoid confounding factors related to the performance of specific implementations, an experimental assessment was conducted using “ideal” solutions. The results, collected from 10 subjects, demonstrated clear differences between the SA modalities vs. the manual baseline (volitional control only) and across the SA modalities in performance, volitional control usage, and reaction time. The differences were exacerbated when systematic errors were introduced in the autonomous control.

In the no-error condition, the subject and the autonomous agent had similar goals (the same *desired hand configuration*). In this case, the SA modalities were consistently faster than the pure volitional control (manual baseline). Several studies have demonstrated that SA systems can outperform conventional EMG-based controllers (Markovic et al., 2015; Volkmar et al., 2019; Mouchoux et al., 2021). The present study confirmed that this result also holds in the case of “ideal” implementations. This demonstrates the intrinsic potential of the SA approach, which, if reliably implemented, can outperform even *a reliable* myoelectric controller. The autonomous component of the SA system provides simultaneous activation of the DoFs and supports movement planning and execution. However, with a purely volitional approach, the subject generated commands sequentially, and she moved the prosthesis nearer to the object, as well as adjusted the wrist to orientate the hand toward the object. Thus, she managed two tasks, leading to slower forearm movements or sequential handling of these two actions (first reach and then orient). Notably, the volitional controller in the present study was implemented as an “ideal” class selector; it is still to be investigated how SA control would compare to an “ideal” regression-based approach, which could be realized using a joystick type interface providing simultaneous control of multiple DoFs.

A difference between the three SA modalities was found even in the baseline (no error) condition. In this case, the *simultaneous SA* modality showed the worst time performance and the highest usage of volitional control among the schemes. The reason for this might be that the user was required to activate a DoF to trigger the autonomous module to control the other DoFs. In fact, we observed that the participants often generated redundant commands for the prosthesis opening (even when the prosthesis was fully open) to trigger the autonomous controller to adjust the wrist DoFs. Conversely, in the other two SA modalities, the autonomous control was active without triggering, and the subject only had to bring the prosthesis into a graspable position and then close the fingers. Therefore, the longer task completion time suggests that the SA scheme with triggering (i.e., simultaneous SA) negatively affects not only the volitional control usage but also the time performance. While triggering was used as a predominant approach in the studies that implemented SA prostheses, some recent approaches eliminated this step (Mouchoux et al., 2021) and/or attempted to make it transparent and effortless, that is, naturally embedded in the grasping process (Frisoli et al., 2012; McMullen et al., 2014).

When a mismatch occurs between the goals (*desired hand configuration*) of the user and the autonomous agent (e.g., due to the errors introduced), the results indicate that the design of the shared control modality can have a critical impact on the task performance as well as the physical and cognitive workload. This situation can arise because of erroneous decisions of the autonomous system (e.g., wrong estimation of object properties (Markovic et al., 2014; Mouchoux et al., 2021) and/or class (Ghazaei et al., 2017; Hundhausen et al., 2019; Shi et al., 2020) or because the autonomous system associates another grasping strategy with the target object than the one intended by the user. For instance, autonomous systems based on object recognition are object-focused, whereas humans are manipulation-focused (Rosenbaum et al., 1996) while selecting the grasp strategy. When the goals of the two control agents are similar, they cooperate, making the task faster and reducing the interaction between the autonomous controller and the subject. Therefore, in this case, a specific implementation of the shared control modality has a smaller impact on the performance. However, when the decisions differ, the scenario develops into a control paradigm in which a conflict arises between the two independent agents (see Figure 7C). This explains an increase in physical and cognitive workload and the decrease in performance observed in the experiment. This can be so detrimental that SA control becomes substantially worse compared to the manual baseline.

Nevertheless, the results also reveal that some SA modalities are inherently more robust in these situations. The *sequential SA* modality was less impacted by the introduction of errors compared to the two other schemes. When the user noticed the wrong configuration, she engaged in volitional control to correct it. Importantly, in the sequential modality, this also fully disabled the autonomous controller. Therefore, the system gracefully “degraded” to a pure volitional control; indeed, the outcome measures for *sequential* modality in error conditions were similar to those of the manual baseline. Therefore, a *sequential* approach might be the modality of choice, especially because an SA prosthesis is used in dynamic and challenging environments (e.g., at work).

On a more general level, the present study is related to the broader field of human-robot collaboration (Cherubini and Navarro-Alarcon, 2021). The quality of shared control depends on several factors listed by Flemisch et al. (2016), such as the traceability and predictability of abilities and intents in both directions (human-machine and machine-human) as well as the arbitration of conflicts. The importance of considering the potential conflict has also been stressed by Abbink et al. (2018): “*In shared control, conflicts between the human and the robot should be minimized, by modeling robot actions based on human behavior; and in case of conflicts, the robot should ensure that the human has the time and ability to influence the robot's actions*.” The current results demonstrated that these points impact the performance, physical, and cognitive workload and need to be addressed while designing a collaborative control (Sherstan et al., 2015). One solution already tested in prosthetics is to check for concordance between the commands of the two agents and prioritize the user's commands in case of any conflicts (Zhuang et al., 2019). Abbink et al. (2018) stated that “*the control authority can be traded with enough margins for the human operator to get back in the loop and respond adequately,”* which is in line with better performance obtained by using *sequential SA* modality in the present study.

The data collected in this experiment were obtained from able-bodied users. Their relation and experience with the prosthesis are different from those of an amputee relying on a prosthesis in daily life. Not only may the control priorities differ, but the perception of the autonomous system may also be different. Indeed, the embodiment of a prosthesis is a relevant parameter of its acceptance and use (Fritsch et al., 2021). Therefore, it would be interesting to complement the quantitative outcomes measured in this study with the effect that the shared control modality might have on the prosthesis's embodiment in the user's self-representation. Furthermore, the present experiment cannot be directly reproduced in amputee subjects, as the volitional control module uses mechanical switches to achieve intuitive and reliable control (“ideal” implementation). The aim of the present study was not to develop a novel SA system, but to investigate the fundamental impact of different “prototypical” SA schemes on the interaction between the system and its user. To maintain the intuitiveness for amputee subjects, the volitional controller would need to rely on the pattern classification of myoelectric signals. Nevertheless, in this case, the reliability of the control could be improved through a more extensive training. Finally, although the study design introduced substantial within-task variability by combining random perturbations in prosthesis orientation with different object grasping directions, the overall variability of tasks was limited by the fact that the employed objects were of similar size and function. User's interaction with an object greatly depends on its (intended) application; therefore, in order to better understand their strengths and weaknesses, the SA control-sharing modalities (and corresponding SA systems) will need to be evaluated using an extended set of objects that are common in activities of daily living. Such investigation is however outside the scope of the present work and remains a future goal.

## Conclusion

This study investigated the impact of shared control modalities on performance and workload while using a SA prosthesis. The results indicate that all SA modalities outperformed pure volitional control under ideal conditions. However, *simultaneous* schemes were worse than the sequential and continuous modalities. When the accuracy of the autonomous controller degraded (error conditions), the performance of the SA modalities decreased substantially, even below the manual baseline. However, the most robust approach was the sequential scheme wherein the subject control completely disabled the autonomous controller. This implies that such a scheme is likely the method of choice while implementing upper-limb prostheses equipped with SA control.

## Data Availability Statement

The raw data supporting the conclusions of this article will be made available by the authors, without undue reservation.

## Ethics Statement

The studies involving human participants were reviewed and approved by Ethical Committee of the University Medical Center Göttingen. The patients/participants provided their written informed consent to participate in this study.

## Author Contributions

JM, MABC and MM conceptualized the study. JM and MABC developed the system and wrote the manuscript draft. JM conducted the experiment and performed the data analysis. SD, AS, and MM contributed to the study design and revised the manuscript. All authors approved the submitted version.

## Funding

This work was supported by the German Ministry for Education and Research (BMBF) under the project INOPRO (16SV7657) and in part by the Independent Research Fund Denmark (ROBIN, #8022-00243A).

## Conflict of Interest

The authors declare that the research was conducted in the absence of any commercial or financial relationships that could be construed as a potential conflict of interest.

## Publisher's Note

All claims expressed in this article are solely those of the authors and do not necessarily represent those of their affiliated organizations, or those of the publisher, the editors and the reviewers. Any product that may be evaluated in this article, or claim that may be made by its manufacturer, is not guaranteed or endorsed by the publisher.
